# Blood donation barriers and facilitators of Sub‐Saharan African migrants and minorities in Western high‐income countries: a systematic review of the literature

**DOI:** 10.1111/tme.12517

**Published:** 2018-03-01

**Authors:** E. F. Klinkenberg, E. M. J. Huis In't Veld, P. D. de Wit, A. van Dongen, J. G. Daams, W. L. A. M. de Kort, M. P. Fransen

**Affiliations:** ^1^ Department of Donor Studies Sanquin Research Amsterdam The Netherlands; ^2^ Department of Public Health Academic Medical Center, University of Amsterdam Amsterdam The Netherlands; ^3^ Department of Medical and Clinical Psychology Tilburg University Tilburg The Netherlands; ^4^ School of Psychology, University of New South Wales Sydney Australia; ^5^ Medical Library Academic Medical Center, Univeristy of Amsterdam Amsterdam The Netherlands

**Keywords:** Africa south of the Sahara, African migrant, blood type, ethnic minorities, inheritable blood disorder, motivators, needle fear, personal discrimination

## Abstract

**Objectives:**

The present study aimed to gain more insight into, and summarise, blood donation determinants among migrants or minorities of Sub‐Saharan heritage by systematically reviewing the current literature.

**Background:**

Sub‐Saharan Africans are under‐represented in the blood donor population in Western high‐income countries. This causes a lack of specific blood types for transfusions and prevention of alloimmunisation among Sub‐Saharan African patients.

**Methods/materials:**

Medline, EMBASE, PsycINFO and BIOSIS were searched for relevant empirical studies that focused on barriers and facilitators of blood donation among Sub‐Saharan Africans in Western countries until 22 June 2017. Of the 679 articles screened by title and abstract, 152 were subsequently screened by full text. Paired reviewers independently assessed the studies based on predefined eligibility and quality criteria.

**Results:**

Of the 31 included studies, 24 used quantitative and 7 used qualitative research methods. Target cohorts varied from Black African Americans and refugees from Sub‐Sahara Africa to specific Sub‐Saharan migrant groups such as Comorians or Ethiopians. Main recurring barriers for Sub‐Saharan Africans were haemoglobin deferral, fear of needles and pain, social exclusion, lack of awareness, negative attitudes and accessibility problems. Important recurring facilitators for Sub‐Saharan Africans were altruism, free health checks and specific recruitment and awareness‐raising campaigns.

**Conclusion:**

The findings of this review can be used as a starting point to develop recruitment and retention strategies for Sub‐Saharan African persons. Further research is needed to gain more insight in the role of these determinants in specific contexts as socioeconomic features, personal histories and host country regulations may differ per country.

In many Western countries, minority populations (such as immigrants and refugees but also individuals with total or partial ancestry from non‐White racial groups) are under‐represented in the blood donor population (Murphy *et al.,* 2009; Rastogi *et al.,* 2011). Certain specific blood types are more common in certain ethnic groups than others, especially among those of Sub‐Sahara African (SSA) background (Reid *et al.,* 2002). For instance, the Duffy negative phenotype (Fy(a‐b‐)) is frequently found in the Sub‐Saharan region of Africa but is rarely present among individuals in countries consisting largely of White European‐origin people (Howes *et al.,* 2011).This discrepancy in blood types poses a problem because, if donor blood and patient blood do not match well, serious complications can occur (Yazdanbakhsh *et al.,* 2012), such as haemolytic transfusion reactions caused by the development of antibodies in response to antigens in donor blood (Miller *et al.,* 2013). Patients in need of repeated blood transfusions are especially at a high risk of alloimmunisation. One example is sickle cell disease (SCD), a relatively common inheritable blood disorder among SSA individuals (Rees *et al.,* 2010). Many patients with SCD who receive red blood cells produce antibodies and are thus alloimmunised (Miller *et al.,* 2013; Alkindi *et al.,* 2017). An adequate supply of well‐matched, antigen‐negative red blood cells is needed to improve the blood supply and to enable helping patients with an SSA background. This makes SSA individuals an important target group for blood donation agencies (van Dongen *et al.,* 2016).

Unfortunately, blood agencies all over the world have problems recruiting SSA blood donors (Grassineau *et al.,* 2007; Shaz & Hillyer, 2010a). In part, this is attributable to existing regulations in some countries, such as the exclusion of individuals with language barriers and SCD and Thalassemia carriers (van Dongen *et al.,* 2016). On the other hand, attempts to recruit healthier SSA donors have fallen short, or some recruitment programmes seem to appeal to the majority population only (Frye *et al.,* 2014; Muthivhi *et al.,* 2015). To optimise recruitment and retention strategies, more insight is needed on what prevents and motivates people of an SSA background to donate blood.

Recent systematic reviews of the literature have focused on SSAs in their birth countries rather than on those living as ethnic minorities or migrants in Western countries (Tagny *et al.,* 2010; Burzynski *et al.,* 2016). According to the qualitative syntheses in these systematic reviews, health‐ and knowledge‐related barriers are commonly cited by SSAs. More specifically, there is a fear of being exposed to various infectious diseases (Burzynski *et al.,* 2016), but there is also a high prevalence of transmissible infections among blood donors, which impacts blood safety (Tagny *et al.,* 2010). Replacement/family donations are also predominant in SSA countries instead of voluntary non‐remunerated donations. Due to the different blood donation and supply systems between SSA countries and Western countries, the barriers and facilitators experienced may differ. Earlier studies regarding barriers and motivators of SSAs in non‐African countries were summarised but have not been systematically reviewed before (Shaz *et al.,* 2008; Shaz & Hillyer, 2010a). In addition, these summaries focused only on African Americans (AAs) in the United States but not on other countries where their blood is needed, such as Australia or European countries.

A better understanding on what prevents and motivates potential SSA blood donors in different Western countries to donate blood would allow the development of more effective recruitment and retention strategies. The present study aimed to gain insight into the barriers/facilitators of blood donation among SSAs in high‐income countries where the majority were White or Caucasian and into differences between SSA and White individuals by systematically searching and analysing the current literature.

## METHODS

### *Search strategy*

**Figure 1:**
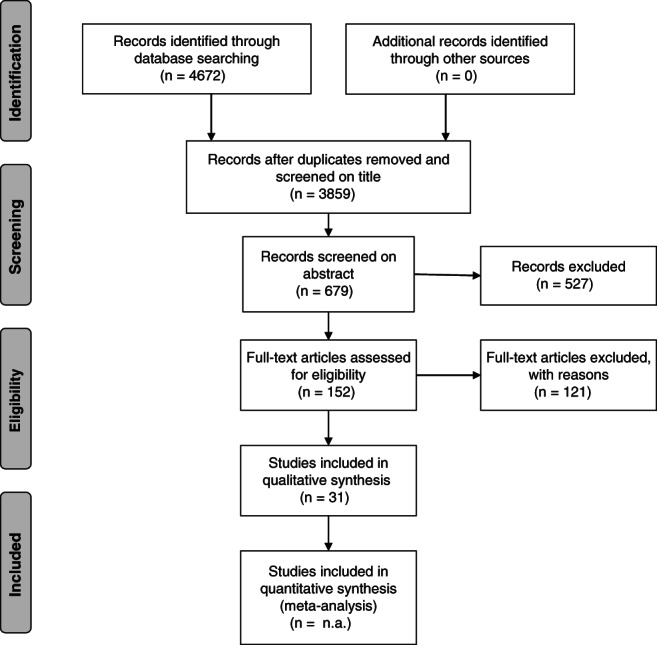
Flow diagram for this systematic review on qualitative and quantitative studies exploring the experienced or reported barriers/facilitators for donating blood among African minorities in White majority countries. Adapted from Moher *et al.* (2015).

Medline, EMBASE and PsycINFO were systematically searched for articles or abstracts published from inception until the 22nd of June 2017. BIOSIS was searched until the 19th of October, 2015, due to the discontinued licence of the database. The search resulted in a total of 4672 articles (Medline, *N* = 776; EMBASE, *N* = 1853; PsycINFO, *N* = 1596; BIOSIS, *N* = 447). No additional relevant articles were identified through manual searching of other sources (*n* = 0). After removing duplicates, 3859 articles were screened on initial relevance based on the title, and the resulting 679 articles were screened by title and abstract. Of the resulting 152 articles screened by full text, 121 were excluded based on the eligibility criteria, thus leaving 31 articles for the present quality assessment (Fig. 1) (Moher *et al.,* 2015).

An initial scoping of the literature led to the identification of three relevant search concepts: [blood donation] AND [race, minorities and ethnicity] AND [factors – barriers & facilitators]. For each concept, relevant (controlled) terms were employed. Animal studies were excluded. Appendix A presents details for each database.

### *Eligibility criteria*

We included studies if they explicitly focused on possible barriers and facilitators that may influence blood donation behaviour and intention among adults (about 18–65 years) of SSA origin or background living in a high‐income country with a White European or Caucasian majority. The possible barriers and facilitators could be either experienced or self‐reported and could refer to factors either negatively or positively associated with blood donation behaviour, blood donor status or intention to donate or become a blood donor. Both descriptive studies on SSA minorities or migrants only and comparative studies with White or other subgroups were included.

SSAs were defined as individuals who originated from countries lying south of the Sahara Desert in Africa. In American studies, those of African ancestry are commonly referred to as Blacks or AAs. Although the precise definition of these labels is unclear, most AAs came to the United States during the Colonial era. We decided to include these latter studies as the terms are commonly used for persons who originate from West or Central Africa and are, thus, carriers of blood types not common in the White European or Caucasian population and are an important target population for blood donor recruitment and retention (Reiner *et al.,* 2011).

Only empirical studies were included: quantitative questionnaire or database results and qualitative interview or focus group results. We excluded case reports, reviews and viewpoints. Studies in countries where whole blood donors are remunerated in cash for their whole blood donations are excluded, as well as studies that are solely on other types of donation (e.g. organs, platelets).

### *Quality assessment*

We created two quality criteria lists for quality assessment of the quantitative and qualitative studies (Appendices B and C). They included items from different quality assessment tools, thus creating comprehensive lists to assess the risk of bias in the varying designs of the studies. The Critical Appraisal Skills Programme (CASP) (Singh, 2013), the STROBE statement (Von Elm *et al.,* 2007), the QualSyst tool (Kmet *et al.,* 2004) and the Critical Review Form for Quantitative Studies (Law *et al.,* 1998) provided quality criteria for the quantitative studies. The CASP (Singh, 2013), the QualSyst tool (Kmet *et al.,* 2004), the Consolidated Criteria for Reporting Qualitative Studies (COREQ) (Tong *et al.,* 2007), the modified quality checklist used by Mills *et al*. (2005) and the Cochrane risk of bias tool (Offringa *et al.,* 2003) provided quality criteria for the qualitative studies. For each quality criteria list, two authors scored each article and compared each other's assessment and resolved differences. All items were weighed equally for the overall quality score. Similar methods and score systems were used in previous systematic reviews of the literature (Hoogerwerf *et al.,* 2015; Piersma *et al.,* 2017).

## RESULTS

### *Characteristics of the included studies*

The characteristics of the quantitative studies are presented in Table 1 and the characteristics of the qualitative studies in Table 2. All included studies were published between 2002 and 2016. Most were conducted in the United States (*n* = 21), followed by Australia (*n* = 5) and Canada (*n* = 2). The remaining three studies were conducted in Israel (*n* = 1), the UK (*n* = 1) and France (*n* = 1). All Australian studies, as well as the two Canadian studies, were conducted by the same research group in each country with recurring authors. The Australian quantitative studies used the same data (425 migrants and refugees from Africa), as did the Australian qualitative studies (88 migrants and refugees from Africa). In the United States, 16 of the 21 studies were conducted by recurring (groups of) authors. Both the studies by Boulware *et al*. used the same data (385 individuals from households in Maryland, USA) (Boulware *et al.,* 2002a,b).

**Table 1:** Characteristics of the quantitative studies (*n* = 24)

Study	Country	Objective/aim	Design	Participants	Main barriers/facilitators
1. Amponsah‐Afuwape *et al*. (2002)	UK	Investigate blood donation intention among ethnic minorities using the Theory of Planned Behaviour.	Questionnaires in university eateries and libraries.	Asian (*n* = 38), Black (*n* = 42) and White (*n* = 66) high‐school students.	Barriers → In‐group altruism and ethnic group identification.
2. Boulware *et al*. (2002b)	USA	Study the contribution of sociodemographic, medical and attitudinal factors in explaining likelihood to donate blood.	Telephone survey	Maryland households (*n* = 385)	Barrier → Fear of hospitals.
3. Boulware *et al*. (2002a)	USA	Studying which factors are most important in explaining race and gender disparities in willingness to donate	Telephone survey	Maryland households (*n* = 385)	Barriers → Mistrust of hospitals and concerns about discrimination.
4. Cable *et al*. (2011)	USA	Evaluate the effects of blood donation intensity on iron and haemoglobin deferral in a prospective study	Self‐administered questionnaire, donor and deferral databases.	Whole blood or double red blood cell donors 18 years or older (*n* = 2425).	Barrier → Hb deferral.
5. Custer *et al*. (2012)	USA	Investigate the demographic characteristics of successful, unsuccessful and deferred donor visits over a 4‐year time period	Donor and deferral databases.	Donor presentations (*n* = 5 607 922).	Barrier → Haematocrit/Hb deferral.
6. Glynn *et al*. (2002)	USA	Evaluate reasons to donate, influencing factors and potential responses to a variety of reminders in whole blood donors.	Survey via e‐mail	45 588 allogeneic whole blood donors	Facilitators → Receiving an item/gift and receiving infectious disease test results.
7. Glynn *et al*. (2006)	USA	Evaluate the role of various potential motivators in the decision to donate of first‐time and repeat Asian, Hispanic, Black and White whole blood donors.	Web‐based questionnaire.	7922 whole blood donors	Facilitators → Appeal or request by work, rewards, gifts, time of work, health screens, enjoy helping others and feeling pressured.
8. Grossman *et al*. (2005)	USA	Assess potential barriers and motivators to blood donation among African American women.	Telephone survey	162 African American women from St. Louis.	Barriers → Too inconvenient, afraid of needles, takes too much time and concerned about contracting a disease. Facilitators → Increase awareness of need, more convenient locations and encouragement by pastor.
9. James *et al*. (2011)	USA	Evaluate whether mistrust for the healthcare system among African Americans affects attitudes towards blood donation.	Self‐administered questionnaire	930 individuals from African American religious institutions in Atlanta.	Barriers → Rarely think about it, afraid to give blood, afraid of needles, pain or discomfort, afraid of feeling faint, dizzy, or unwell and mistrust in hospitals. Facilitators → Help save a life, it is the right thing to do, help the community and because blood is needed.
10. James *et al*. (2012)	USA	Studying the prevalence of blood donor eligibility factors among different demographic groups.	Multiple data sources	185 073 489 individuals aged between 18 and 65 years.	Barriers → Low Hb and HBV infection deferral.
11. James *et al*. (2013)	USA	Investigate factors that serve as motivators and barriers to blood donation among AA and Western individuals.	Mailed survey to registered voters in Atlanta	281 registered voters aged between 18 and 69 years.	Barriers → No convenient place to donate, now knowing where to donate, and afraid of needles, pain or discomfort. Facilitators → More convenient place to donate, assurance that donating is safe, more convenient times to donate.
12. James *et al*. (2014)	USA	Geographic analysis to blood donor behaviour and use of different donation sites.	Database of American Red Cross Blood Services, Southern Region	402 692 blood donors in Georgia with 1 147 442 blood units.	Barrier → Geographical barriers (travel distances, lack of donation sites in minority communities).
13. Mast *et al*. (2010)	USA	Better understand the underlying causes of low Hb deferral.	Donation and deferral database	715 311 unique donors	Barrier → Hb deferral.
14. McQuilten *et al*. (2014)	Australia	Determine the proportion of African migrants who had previously donated blood, and what sociodemographic factors are associated with donation.	Cross‐sectional surveys by bilingual interviewers	425 African migrants and refugees living in Victoria	Facilitator → High blood donation knowledge.
15. Merav & Lena (2011)	Israel	Examining whether the Theory of Planned Behaviour adds significantly to the prediction of intention and actual blood donation of the general Israeli population.	On‐site questionnaires in central Pardes Hanna	Native Israelis (*n* = 75) and Ethiopian Israelis (*n* = 51)	Barriers → Afraid the donated blood is not used, decisions on not using blood is made on a non‐medical basis and finding important how the blood is used.
16. Polonsky *et al*. (2013)	Australia	Examine the applicability of the basic TPB model, and extend the TPB model with overall knowledge of blood donation.	Cross‐sectional surveys by bilingual interviewers.	425 African migrants and refugees living in Melbourne and Adelaide (Victoria).	Facilitator → Blood donation knowledge.
17.Renzaho & Polonsky (2013)	Australia	Assessing whether perceived discrimination, acculturation and medical mistrust are associated with knowledge about blood donation and blood donation status.	Cross‐sectional surveys by bilingual interviewers.	425 African migrants and refugees living in Melbourne and Adelaide (Victoria).	Barrier → Perceived discrimination.
18. Schreiber *et al*. (2006)	USA	Identify barriers and factors that can be effectively addressed by blood centres.	Self‐administered survey in 6 American blood centres	4142 lapsed whole blood donors.	Barriers → No convenient place to donate, changed jobs and poor staff skill.
19. Shaz *et al*. (2009a)	USA	Determine specific motivators and barriers to blood donation for AA individuals.	Online survey via e‐mail.	364 participants from two historically African colleges/universities in southeastern USA.	Barriers → Feeling faint, dizzy or nauseated and concerns about the safety. Facilitators → convenient place, university involvement in promoting blood drives and feeling of self‐satisfaction.
20. Shaz *et al*. (2009b)	USA	Determine differences in motivators and barriers between AA and Western current blood donors.	Self‐administered questionnaire at fixed donation sites.	598 blood donors from two different donation centres.	Facilitators → Help save a life, being treated well by the staff and being called to donate when there is a shortage.
21. Shaz *et al*. (2010c)	USA	Evaluate donor deferral rates and the reasons for deferrals by race, gender and age in a metropolitan area.	Donor screening, questionnaire and database	Donor presentations between 2004 and 2008 and aged 16–69 years (*n* = 576 317).	Barrier → Hb deferral.
22. Shaz *et al*. (2010b)	USA	Identify motivators and barriers to African Americans donating blood.	Self‐administered questionnaire at predominantly African American religious institutions in Atlanta.	930 respondents from 15 African American churches (99% African American).	Barriers → Inconvenient location/times, rarely thinking about it, being afraid, nervous or anxious. Facilitators → Help save a life, help the community and because blood is needed.
23. Steele *et al*. (2012)	USA	Evaluate differences in knowledge and beliefs about AIDS by demographics and by donor status.	Telephone interview of general US population.	*n* = 9859	Barrier → Concerns about safety (regarding to AIDS).
24. Vahidnia *et al*. (2016)	USA	Understand motivating factors that contribute to the decision to donate blood for infected and uninfected blood donors	Interviewer‐administered telephone or in‐person questionnaires	1002 infected donors and 1387 control donors	Barriers → test seeking and negative attitude (towards screening policies)

**Table 2:** Characteristics and quality assessment of the qualitative studies (*n* = 7)

Study	Country	Objective/aim	Design	Participants	Relevant results
1. Charbonneau & Tran (2013)	Canada	Examine blood's representations in Quebec.	Semi‐structured qualitative interviews	*n* = 234, from which 76 were minority informants.	Facilitator → Donating within the community.
2. Frye *et al*. (2014)	USA	Describe the implementation and evaluation of the Precise Match programme.	Documentation of programme implementation, focus group results and data on donations.	n/a	Barriers → Hb deferral, fear, and distrust. Facilitators → Presenting needy recipients, representatives from diverse ethnic communities.
3. Grassineau *et al*. (2007)	France	Present the method used in a blood drive to promote blood collection in a SSA migrant community formed by Comorians living in Marseilles.	Semi‐structured qualitative interviews and setting up a community‐action group.	Comorian immigrants (*n* = 59)	Barriers → distrusting use of blood, infectious disease markers, conceptions about blood inside the community.
4. Mathew *et al*. (2007)	USA	Understanding barriers and motivators of blood donation and evaluate whether these differ between demographic groups.	Six focus groups	Donors or potential donors in the Washington, DC, suburbs aged 18–65 years (*n* = 53).	Barriers → Fear, inconvenience and lack of awareness. Facilitators → Target the specific needs of minority communities, creating convenience and educational campaigns.
5. Polonsky *et al*. (2011a)	Australia	Ascertain whether the way wider society views African migrants, impacts on migrants' desire to donate blood and their perceived level of social inclusion.	Nine semi‐structured group discussions	88 migrants and refugees from African countries.	Barriers → Discrimination, marginalisation and social exclusion. Facilitator → Altruism and acknowledgement.
6. Polonsky *et al*. (2011b)	Australia	Examine the degree to which home and host country beliefs enable and/or deter blood donation among African communities in Australia.	Nine semi‐structured group discussions	88 migrants and refugees from African countries.	Barriers → Lack of knowledge, mistrust and discrimination. Facilitators → Need of blood and saving a life.
7. Tran *et al*. (2013)	Canada	Explore blood donation among Black communities in a sociocultural context.	Semi‐structured qualitative interviews	African donors (*n* = 10), African community leaders (*n* = 17), and blood agency personnel (*n* = 6).	Barriers → Perceived discrimination and social exclusion. Facilitators → increased awareness about sickle cell anaemia and the importance of their contribution.

### *Quality descriptives and issues*

Tables 3 and 4 present an overview of the quality criteria and the scores for the quantitative studies and the qualitative studies, respectively. A total score of 100% means that the study meets all criteria, whereas a score of 0% would mean that the study meets none of the criteria. Almost all quantitative studies addressed a clearly focused issue and described specific objectives, and all qualitative studies provided a clear aim of the study. However, we encountered many methodological issues for both the quantitative and qualitative studies. For the quantitative studies, the study sample was often not representative of a defined population, or it was not sufficiently explained why this particular sample was chosen or necessary to study. In addition, the response rate and characteristics of the study sample were often not mentioned, and many studies did not control for possible confounders, which are partly due to the descriptive, rather that analytical, approach of many studies. Regarding the methodological issues of the qualitative studies, the role of the researcher was only discussed in two of the seven studies. The researchers' own ethnic and cultural background may be a potential bias, especially in studies on ethnic communities. Besides, the locations of the interview/focus groups were often not described, and for almost half of the studies, it remained unknown whether the researchers had taken ethical issues into consideration.

**Table 3:** Overview of the quality scores for the quantitative articles (*n* = 23)

Study	1. Focus	2. Objectives	3. Design	4. Recruitment	5. Variables	6. Analysis	7. Results	8. Discussion	Score
1. Amponsah‐Afuwape *et al*. (2002)	+	+	+/−	−	+/−	+/−	+/−	+/−	56%
2. Boulware *et al*. (2002b)	+	+	+	+/−	+	+/−	+	+/−	91%
3. Boulware *et al*. (2002a)	+	+	+	+/−	+	+/−	+/−	+/−	81%
4. Cable *et al*. (2011)	+	+	+	+/−	+/−	+/−	+	+/−	75%
5. Custer *et al*. (2012)	+	+	+	+	+/−	+/−	+/−	+/−	84%
6. Glynn *et al*. (2002)	+	+/−	+/−	+	+/−	+	+	+	88%
7. Glynn *et al*. (2006)	+/−	+	+	+/−	+/−	+/−	+	+/−	78%
8. Grossman *et al*. (2005)	+	+	+/−	+/−	−	+/−	+/−	+/−	56%
9. James *et al*. (2011)	+	+	+/−	+/−	+/−	+/−	+/−	+/−	66%
10. James *et al*. (2012)	+	+	+	+	+	+	+/−	+	84%
11. James *et al*. (2013)	+	+/−	+/−	+/−	+/−	+/−	+/−	+/−	59%
12. James *et al*. (2014)	+	+	+	+	+	+	+	+	100%
13. Mast *et al*. (2010)	+	+	+	+	+	+/−	+	+/−	91%
14. McQuilten *et al*. (2014)	+	+	+	+/−	+/−	+/−	+/−	+/−	81%
15. Merav & Lena (2011)	+	+/−	+/−	+/−	+/−	+/−	+/−	+/−	63%
16. Polonsky *et al*. (2013)	+	+	+	+/−	+	+/−	+/−	+/−	81%
17.Renzaho & Polonsky (2013)	+	+	+/−	+/−	+/−	+/−	+/−	+	72%
18. Schreiber *et al*. (2006)	+	+	+/−	+/−	+/−	+/−	+/−	+/−	69%
19. Shaz *et al*. (2009a)	+/−	+	+/−	+/−	+/−	−	+/−	+/−	50%
20. Shaz *et al*. (2009b)	+	+	+/−	+/−	+/−	+/−	−	+/−	53%
21. Shaz *et al*. (2010c)	+/−	+	+/−	+	+/−	−	+/−	+/−	69%
22. Shaz *et al*. (2010b)	+	+	+	+/−	+/−	−	+/−	+/−	66%
23. Steele *et al*. (2012)	+/−	+/−	+	+/−	+/−	+	+	+/−	75%
24. Vahidnia *et al*. (2016)	+	+	+	+/−	+/−	+	+	+/−	81%

+ Fully meets the criterion; +/− Partly meets the criterion; − Does not meet the criterion.

**Table 4:** Overview of the quality scores for the qualitative articles (*n* = 7)

Study	1. Aim	2. Design	3. Theory/knowledge	4. Recruitment	5. Data collection	6. Findings	7. Value of study	Score
1. Charbonneau & Tran (2013)	+	+	+	−	+/−	+	+/−	75%
2. Frye *et al*. (2014)	+	+/−	+/−	−	+/−	+/−	−	50%
3. Grassineau *et al*. (2007)	+	+/−	+	+/−	+/−	+/−	−	50%
4. Mathew *et al*. (2007)	+	+	+/−	+/−	+/−	+/−	+	79%
5. Polonsky *et al*. (2011a)	+	+/−	+	+/−	+	+	+/−	86%
6. Polonsky *et al*. (2011b)	+	+	+	+/−	+	+	+	96%
7. Tran *et al*. (2013)	+	+	+	+/−	+	+	+	96%

+ Fully meets the criterion; +/− Partly meets the criterion; − Does not meet the criterion.

## BARRIERS TO BLOOD DONATION

### *Lack of knowledge and awareness*

McQuilten *et al*. (2014) found African migrants and refugees with moderate blood donation knowledge to have an almost 4·5 times higher odds on having donated previously compared to those with poor knowledge (adjusted odds ratio, AOR [95% confidence interval, CI] = 4·46 [1·57–12·67]; *P* < 0·01). For those with a high level of knowledge, the odds were more than 10 times higher compared with those who had poor knowledge [*AOR* (95% CI) = 11·30 (3·79–33·70); *P* < 0·001]. In addition, Polonsky *et al*. (2013) found that adding knowledge to the original Theory of Planned Behaviour (TPB) model increased the model fit for SSAs. The TPB is a commonly used theory in blood donor studies, whereas attitudes, social norms and self‐efficacy predict the intention and behaviour to donate blood (Ajzen, 1991; Lemmens *et al.,* 2005). However, James *et al*. (2011) found AAs to have a fairly good knowledge of blood donation and that there were no differences in the scores between AA donors and AA non‐donors. In addition, Renzaho & Polonsky (2013) found marginalisation to be negatively related to blood donation knowledge, but there was no evidence that marginalisation was related to actual blood donation.

Concerning the lack of awareness, for both AA donors and AA non‐donors, not knowing that donating blood is important (23·1% donors; 21·8% non‐donors) and not knowing where to donate (23·9% both donors and non‐donors) were important self‐reported barriers (Shaz *et al.,* 2010b). There was evidence that AAs from the general population in Atlanta, Georgia, more often did not know where to donate compared with White individuals (AA 31%, White 19%) (James *et al.,* 2013). In the qualitative study by Polonsky *et al*. (2011b), respondents from Australian‐based African communities reported that they had never discussed blood donation or had never been approached about blood donation before their research.

### *Negative attitude*

Schreiber *et al*. (2006) found AA first‐time donors being more likely to report poor staff skills (*P <* 0·01) and experiencing bad treatment (*P <* 0·01) compared with White first‐time donors. The African migrant respondents in Australia in Polonsky *et al*. (2011a) also stated, in interviews, that they experienced poorer treatment and longer waiting times compared with other patients. Accordingly, Ethiopians, compared with native Israelis, had a more negative behavioural attitude towards blood donation [*t*(124) = 4·0, *P <* 0·01] (Merav & Lena, 2011). Lastly, Vahidnia *et al*. (2016) found that AAs are more likely to believe that the screening policies of the blood bank are unfair compared with Whites [AOR (95% CI) = 0·3 (0·1–0·7); *P =* 0·01].

### *Mistrust*

A higher proportion of AAs compared with Whites believed that hospitals wanted to know more about their personal affairs than they needed to know (AA men 48%, AA women 37%, White men 29%, White women 19%; *P* < 0·01) and that hospitals had conducted harmful experiments on patients without their knowledge (AA men 72%, AA women 50%, White men 29%, White women 28%; *P* < 0·01) (Boulware *et al.,* 2002a). Although James *et al*. (2011) found a difference in mistrust between current donors and never donors (AA donor 14%, AA non‐donor 23%), Renzaho & Polonsky (2013) found no such link between African migrants who have ever given blood or have never given blood [odds ratio, OR (95% CI) = 0·98 (0·92–1·03); *P =* 0·42].

Regarding mistrusting the blood supply or donation agencies, Steele *et al*. (2012) found that AAs had more concerns about the safety of blood donation than White individuals, e.g., that not all blood donations were tested for AIDS (acquired immunodeficiency syndrome) [OR (95% CI) = 0·7 (0·6–0·8); *P* < 0·001] and that they could get AIDS from donating blood (43·1% AAs; 15·9% White; *P* < 0·001). AAs were more distrustful towards shortage claims and were more likely to believe that their blood was not wanted and would not be used (Mathew *et al.,* 2007; Merav & Lena, 2011; Tran *et al.,* 2013). In contrast, James *et al*. (2013) found that only 6% of the AAs reported mistrust for blood centres as a barrier.

### *Ethnic discrimination and identification*

Perceived personal discrimination was negatively associated with donating blood in the host country [AOR (95% CI) = 0·63 (0·45–0·86); *P* < 0·01] (Renzaho & Polonsky, 2013). Those who felt discriminated against believed that the general population would not want to receive their blood (Polonsky *et al.,* 2011a). Even experiences of discrimination outside the blood donation setting had a negative impact on AAs' views towards blood donation (Polonsky *et al.,* 2011b). Discrimination was also experienced in healthcare settings where SSAs felt that they were treated worse than others by medical staff (Polonsky *et al.,* 2011a).

Furthermore, several studies found that SSAs would prefer to donate within their own community or, more preferably even, for family members and close acquaintances (Grassineau *et al.,* 2007; Mathew *et al.,* 2007; Charbonneau & Tran, 2013; Tran *et al.,* 2013). Additionally, due to discrimination and social exclusion, these groups preferred to donate blood for their own community rather than for the overall population (Tran *et al.,* 2013). Amponsah‐Afuwape *et al*. (2002) reported ethnic group identification (EGI) and in‐group altruism (IGA) to be negatively related with the intention to donate blood (EGI; *r* = −0·27, *P <* 0*·*01; IGA; *r* = −0·22, *P <* 0*·*01). AAs scored higher on both EGI [*F*(2, 143) = 30·15; *P <* 0*·*001] and IGA [*F*(2, 143) = 40·48, *P <* 0*·*001] compared with Asian and White/European participants.

### *Fear*

Different types of fear were distinguished in the included studies. For instance, AA first‐time donors were significantly more afraid of needles (*P <* 0*·*05) and were more afraid that donating is painful (*P <* 0*·*01) compared with White first‐time donors (Schreiber *et al.,* 2006). The overall prevalence of needle fear ranged from 14 to 38% (Shaz *et al.,* 2009a,b; James *et al.,* 2013). Another type of fear identified in the studies was for fainting. James *et al*. (2013) found White individuals to have a higher prevalence of fear of fainting than AAs (AA 18%, White 29%). Still, fear of fainting is a major barrier for AAs, with a prevalence of 34% among AA non‐donors (Shaz *et al.,* 2010b). Fear of hospitals was also found to be a donation barrier. Those afraid of hospitals had 70% lower odds of prior blood donation compared with those who were not [*OR* (95% CI) = 0·3 (0·1–0·9)] (Boulware *et al.,* 2002b). Lastly, fear of contracting a disease was mentioned by 12% of the AA respondents in the study of Grossman *et al*. (2005) and 22% of the AA respondents in the study of Shaz *et al*. (2010b) but was also commonly mentioned among other ethnic groups (Mathew *et al.,* 2007).

### *Deferral and exclusion factors*

SSAs had the highest chance of haemoglobin (Hb) deferral compared with other ethnic groups (Cable *et al.,* 2011; Custer *et al.,* 2012). While 1·6% of the White men and 16·6% of the White women were deferred for low Hb on their donation attempt, for SSA donors, these rates were 2·4 and 29·2%, respectively (Mast *et al.,* 2010). James *et al*. (2012) found the Hb deferral rate for White persons to be 3·6%, compared with 12% for AA donors. Other commonly reported deferral or exclusion factors for donating blood for SSA donors were: difficulty to find or palpate the veins, high blood pressure or pulse deferral, hepatitis C infections, hepatitis B infections, minor infections (e.g., a cold), tattoos, institutionalisation, pregnancy, cancer, syphilis, malaria, diabetes and cardiovascular problems (Schreiber *et al.,* 2006; Grassineau *et al.,* 2007; Shaz *et al.,* 2010c; Custer *et al.,* 2012; James *et al.,* 2012). These factors cause SSAs to be more often temporarily or permanently deferred for blood donation.

### *Inconvenience*

Six studies found evidence inconvenience to be an important barrier to donate among SSAs. Although most studies focused on an inconvenient location of the donation centre only (*n* = 5) (Grossman *et al.,* 2005; Schreiber *et al.,* 2006; Mathew *et al.,* 2007; James *et al.,* 2013, 2014), one study also took inconvenient opening times into account (Shaz *et al.,* 2010b). From focus group interviews, Mathew *et al*. (2007) found that most individuals felt the opportunities to donate to be limited and that blood centres were not easily accessible. Grossman *et al*. (2005) also found inconvenience to be a common barrier among AA women (19%). AA repeat donors reported inconvenience more frequently (31·4%) compared with White repeat donors (26·3%) (Schreiber *et al.,* 2006). Shaz *et al*. (2010b) found a high prevalence of inconvenience as a barrier, which was 47% for AA current donors and 87% for AA non‐donors. James *et al*. (2014) found that minority communities lacked mobile sites and that these people were thus less likely to donate within their own living area.

## FACILITATORS TO BLOOD DONATION

### *Altruism*

From the studies, we identified different determinants relating to altruistic motivation, such as ‘helping to save a life’ (*n* = 3) (Grassineau *et al.,* 2007;Shaz *et al.,* 2010b ; James *et al.,* 2011) and ‘it is the right thing to do’ (*n* = 4) (Glynn *et al.,* 2002; Shaz *et al.,* 2009b, 2010b; James *et al.,* 2011). In two studies, there is mention of most SSAs strongly agreeing with altruistic motivators, ranging from 63 to 99% (Shaz *et al.,* 2010b; James *et al.,* 2011). However, compared with Whites, SSAs less frequently reported donating because ‘it was the right thing to do’ (AA 77·01%, White 81·80%; *P <* 0·001) (Glynn *et al.,* 2002) (AA 45·2%, White 62·0%; *P* < 0·001) (Shaz *et al.,* 2009b). On the other hand, AA repeat donors were more likely than White repeat donors to donate because they ‘enjoyed helping others’ [*OR* (95% CI) = 1·4 (1·1–1·7); *P <* 0·01] (Glynn *et al.,* 2006). There was evidence of AAs reporting more often of donating to ‘help save a life’ (AA 62·6%, White 47·4%; *P* < 0·01) (Shaz *et al.,* 2009b).

### *Awareness raising/recruitment strategies*

Awareness raising of the importance of blood donation was found to be a regularly mentioned motivator among SSAs (Grossman *et al.,* 2005; Tran *et al.,* 2013). Glynn *et al*. (2002) found that 16·76% of the AA respondents donated because of the appeal of a blood drive organiser or recruiter, which was slightly more than among other ethnic groups (*P* < 0·05). On the other hand, AA donors had the lowest odds of being encouraged by family and friends compared with White donors [*OR* (95% CI) = 0·75 (0·58–0·97); *P* < 0·05]. Glynn *et al*. (2006) found both AA first‐time donors [*OR* (95% CI) = 1·7 (1·4–2·2); *P* < 0·01] and AA repeat donors [*OR* (95% CI) = 1·6 (1·3–1·8); *P* < 0·01] to be more motivated by a request from work to donate blood compared with White first‐time and repeat donors. Shaz *et al*. (2009b) found a larger proportion of AA blood donors than White donors reporting to be motivated by race‐specific marketing campaigns (AA 20·9%, White 3·4%; *P* < 0·001) and community involvement (AA 20·0%, White 4·9%; *P* < 0·001), and Shaz *et al*. (2009a) reported AA students to be motivated by university involvement.

### *Incentives*

Special recognition or awards (donors 11·0%, non‐donors 13·7%) and receiving free gifts (donors 6·3%, non‐donors 9·1%) were the least favourable motivators as reported by AA church attendees (Shaz *et al.,* 2010b). However, James *et al*. (2013) found AAs more frequently reporting to donate for special recognitions or awards (AA 22%, White 11%) and for receiving free gifts (AA 28%, White 17%) than White donors. Glynn *et al*. (2002) found that AAs were more likely to report that they wanted a gift for donating blood compared with White individuals [*OR* (95% CI): 1·40 (1·14–1·72); *P* < 0·01]. Finally, in a later study by Glynn *et al*. (2006), it was found that AA repeat donors were more likely to find gifts [OR (95% CI): 1·4 (1·1–1·9); *P* < 0·01], rewards [*OR* (95% CI): 1·8 (1·3–2·4); *P* < 0·01] and time off work [*OR* (95% CI): 2·1 (1·5–2·9); *P* < 0·01] more important motivators compared with White repeat donors.

### *Health check*

Glynn *et al*. (2002) found that AA donors, compared with White donors, were more frequently in favour of receiving test results for possible infectious diseases (3·26% AA, 2·12% White*; P* < 0·05) (Glynn *et al.,* 2002). Both first‐time AA donors [*OR* (95% CI): 1·9 (1·4–2·4); *P* < 0·01] and repeat AA donors [*OR* (95% CI): 1·6 (1·3–1·9); *P* < 0·01] also had a higher odds compared with White first‐time donors and repeat donors, respectively, to appreciate a health check as an important motivator in the decision to donate blood (Glynn *et al.,* 2006). In coherence with the earlier results, Vahidnia *et al*. (2016) found that AAs were more likely than Whites to report test‐seeking behaviour as a reason to donate blood [*AOR* (95% CI): 2·2 (1·2–3·8); *P =* 0·01].

## DISCUSSION

### *Synthesis of results*

This systematic review indicates that most specific barriers for blood donation in African minority and migrant groups in White/Western majority high‐income countries are: fear of needles, social exclusion, Hb deferral, not being aware of the need, having a negative attitude towards the blood bank policy or organisation and not having a convenient place to donate blood. Fear and a lack of awareness about blood donation are also important and commonly reported barriers for White individuals. White individuals in the included studies also frequently experience Hb deferral and no convenient place to donate blood as important barriers, but there is evidence that these barriers have a bigger impact on SSAs and AAs. For instance, the overall Hb is lower for individuals with an African background (Cable *et al.,* 2011), and blood drives more often visit places with a relatively low proportion of African individuals (James *et al.,* 2014). Lastly, the (perceived) experiences of social exclusion and discrimination are factors that have a large impact on SSA minority groups' intention to donate blood (Renzaho & Polonsky, 2013).

Among the possible facilitators to donate blood in the included studies, we found altruism, health checks and community involvement and campaigns to present promising factors to target in order to facilitate blood donation among SSAs. Altruism was also an important facilitator for White individuals in these studies. There is evidence that SSAs would be more motivated by campaigns focused specifically on (the needs of) their ethnic group and by creating awareness inside their communities.

The barriers and facilitators we found in this review do partly resemble findings from the systematic review by Burzynski *et al*. (2016), which focused on SSAs living in their countries of birth. They too found a lack of knowledge to be a main barrier and helping others to be a main motivating factor. However, although they found health concerns to be an important barrier, in the studies reviewed here, this barrier was not as prevalent. Likewise, although we did find some evidence of SSAs being more concerned with the safety aspect of donating blood, we did not find evidence that a large proportion of the SSAs in Western countries is concerned with a shortage of blood after donating or with adverse health effects to themselves.

### *Limitations*

While most studies reported similar results, some factors yielded mixed results, making the results we found less certain. For instance, the prevalence of medical mistrust differed considerably between the studies: ranging from 14% for AA donors according to James *et al*. (2011) to 72% for AA men according to Boulware *et al*. (2002a). Large differences between studies in percentages for barriers/facilitators were also found for fear, inconvenience and incentives. We speculate that these differences could be attributable to differences in measurements, sample size, sample characteristics of study populations (e.g. students, immigrants, refugees and church members), varying healthcare systems or cultural differences between countries. Most studies originate from the United States and Australia, where the economic and social differences between their racial/ethnic groups are different compared with European countries (OECD, 2015). It remains unknown whether the barriers/facilitators AAs experienced in the United States also apply to SSAs in different continents, especially for the European context. AAs are often descendants of African slaves during the Colonial era and are thus born and raised in the United States, whereas SSAs in European countries are often first‐ or second‐generation immigrants. Arguably, these groups may have different barriers and motivators for donating blood, which we were not able to distinguish, partly due to an under‐representation of studies conducted in Europe. Moreover, some statistically significant differences between SSAs/AAs and White individuals are relatively small in effect sizes or proportions (e.g. for a negative attitude or being motivated by incentives). Therefore, we argue that adjusting recruitment or retention strategies in SSAs regarding these factors – wherever they live – has limited added value.

In addition, only a few quantitative studies used advanced statistical methods, whereas other studies limited themselves to descriptive analyses only. Creating a funnel plot or discussing different effect sizes was deemed impossible because the studies used various research designs. For a more coherent review, it would have been practical to limit the focus to a specific type of design. However, because the main goal of the present study was to explore the barriers/facilitators that are currently studied, we decided to include descriptive studies as well.

### *Implications for practice and research*

We would encourage the development of strategies, in collaboration with African communities, to create more awareness of the need of blood (especially for SCD patients and other patients requiring repeated transfusions, such as patients with haematopoietic disorders). There is evidence that interventions developed for and together with the community are more effective, and this may improve trust in the blood bank organisations (van Dongen *et al.,* 2016). Strategies to reduce barriers for blood donation in this group should focus on investigations on Hb deferral, such as examining possibilities for implementing different reference standards that are still safe for the donor but may reduce deferral rates (Beutler & West, 2005). Finally, the blood bank organisations should contribute to a comfortable environment for SSAs, e.g. by reassuring the blood donors, but also demonstrating what happens with the blood once it is donated. This may contribute to less experienced fear and less mistrust towards the blood bank organisations or their staff.

More research is needed to gain a deepened insight into underlying mechanisms of blood donation among SSAs/AAs. For instance, it would be valuable to more extensively study how specific barriers and facilitators for blood donation actually influence blood donation intention and behaviour. This approach may enable more careful and context‐specific intervention development to increase the chances of implementing more effective recruitment methods. We particularly encourage studies in European countries as most studies are performed in the United States, whereas there is an under‐representation of SSAs in the European blood donor population as well. Although we managed to distinguish important determinants that seem to play a role for Sub‐Saharan minorities in Western high‐income countries, especially the United States and Australia due to the larger amount of studies performed there, the social and personal contexts vary between countries, which may relate to more specific determinants. Future quantitative studies should carefully report the methodology and use statistical hypothesis testing for better generalisability and comparison of results between studies. Measuring the relation between the barriers/facilitators and the donor intention/behaviour would provide more evidence of what kind of interventions may work instead of giving a descriptive overview of the most reported determinants only. In addition, as most results are based on self‐reported barriers and motivators, it may be interesting to look more into the underlying mechanisms of these determinants. For instance, as fear is often reported as an important barrier among SSAs, it would be valuable to monitor whether there are actual differences in levels of stress or anxiety between SSAs and Whites before and after initiating blood donation or seeing a needle. A general overview of possible future research questions based on this systematic literature review can be found in Table 5.

**Table 5:** Summary of literature review findings and recommendations of future research

What is known about this topic?	What new insights does this systematic literature review give?	What are key questions for future work on this topic?
There is quite some research performed already on determinants to donate blood among SSA minorities/migrants. However, an overview of these determinants and an assessment of the quality of these studies are lacking. Therefore, it is unclear which gaps in scientific knowledge exist.	a) This is the first systematic literature review describing the current state of scientific knowledge in blood donation determinants of SSA migrants/minorities in Western high‐income countries. b) By comparing the results of different studies and clustering them in main topics, we found mixed results/small proportions for a lack of knowledge, mistrusting hospitals or blood bank agencies and desiring incentives. c) In the current systematic literature review, the included studies are critically assessed on their quality, which demonstrates that there is profit to be gained in the methodological approaches and descriptions of studies on this topic. There are still gaps in the current literature: d) A majority of these studies do not study the relation between possible determinants and donor intention or behaviour. e) Most results are based on self‐report data. f) Almost no research is published regarding this topic in a European context/country.	a and b) Which barriers/facilitators are good candidates to tackle for blood donor recruitment and retention strategies among SSA migrants/minorities and how? c) — d) How do blood donation barriers/facilitators relate to the intention or actual behaviour to donate blood? e) What are possible underlying mechanisms for blood donation intention or behaviour among SSAs, explaining the main barriers/facilitators? f) What are the main barriers/facilitators of SSA minorities/migrants to donate blood in Europe and how does this compare between European countries, and with minorities/migrants in other continents?

## CONFLICT OF INTEREST

The authors have no competing interests.

## Supporting information


**Appendix A.** Full database search.
**Appendix B.** Quality criteria and full assessment of quantitative studies.
**Appendix C.** Quality criteria and full assessment of qualitative studies.
