# Supplementary material for: Blood donation barriers and facilitators of Sub‐Saharan African migrants and minorities in Western high‐income countries: a systematic review of the literature
Source: Transfus Med. 2018 Mar 1;29(Suppl 1):28–41. doi: 10.1111/tme.12517 (PMC7379919; doi:10.1111/tme.12517)
Supplement: Supplementary file 1 — Appendix A. Full database search. Appendix B. Quality criteria and full assessment of quantitative studies. Appendix C. Quality criteria and full assessment of qualitative studies. [file TME-29-28-s001.docx]

Appendix A
*Full database search*

**20151019 Medline**

blood donors/

(blood adj2 don*).ab,kf,ti.

1 or 2 [blood donation]

ethnic groups/ or "Transients and Migrants"/ or exp African Continental Ancestry Group/ or minority health/ or minority groups/

(race or racial or ethnic* or ethno* or transients or migrant? or minorities or minority group? or cultural sensitivity).ab,kf,ti.

((descent or descendant or heritage or origin? or communit*) adj5 (subsaharan or "sub saharan" or african or "south american" or carribean or non european)).ab,kf,ti.

(non white? or nonwhite? or nonwestern or non western or nonenglish or non english or blacks or afro or afroamerican*).ab,kf,ti.

"Emigrants and Immigrants"/ or refugees/ or "emigration and immigration"/

(refugee? or immigrant?).ab,kf,ti.

or/4-9 [race, minorities and ethnicity]

cultural characteristics/ or cultural diversity/ or exp superstitions/ or taboo/ or health policy/ or symbolism/ or exp culture/ or health literacy/ or exp family relations/ or education/ or exp motivation/ or "attitude to health"/ or patient participation/

(behavio?r or motivation or psychol* or psychiatr* or attitude? or altruism or volunteer* or representation? or barrier? or facilitator? or antecedent? or belief? or value? or norm? or intention* or self efficacy or willing* or unwilling* or taboo? or cultural divers* or cultural characteristic? or superstition? or policy or policies or distance or deterrent? or environmental factor? or recruitment or retention or recruitment or retention or external factor* or determinant? or myth? or folklore or symbolism or location or literacy or (ear* adj3 (experience or history)) or demographic or family or knowledge or education or motivation or demotivation or socio* or accessib* or inaccessib* or trust* or distrust* or mistrust* or relig* or faith or believe? or stigma or discriminat* or aware* or anxiety or fear).ab,kf,ti

identity.ab,kf,ti AND psychology.fs

((self or social or cultural or donor?) adj2 identity).ab,kf,ti

((perspective? or perception?) adj5 (patient? or donor? or donation or respondent? or participant? or client?)).ab,kf,ti

(increas* adj3 (donor? or donation)).ab,kf,ti

or/11-16 [factors]

3 and 10 and 17

animal/ not human/

18 not 19

..dedup 20

omgevingsfactoren:

policy or policies or distance or deterrent? or environmental factor?

===============

**20151019 Embase**

*blood donor/

(blood adj2 don*).ab,kw,ti.

1 or 2 [blood]

exp ethnic group/ or exp Ancestry Group/ or *minority health/ or *minority group/ or *cultural sensitivity/

(race or racial or ethnic* or ethno* or transients or minorities or minority group? or cultural sensitivity).ab,kw,ti.

((origin? or communit* or descent or descendant or heritage) adj5 (subsaharan or "sub saharan" or african or south american or carribean or non european)).ab,kw,ti.

(non white? or nonwhite? or nonwestern or non western or nonenglish or non english or blacks or afro or afroamerican*).ab,kw,ti.

exp migrant/ or exp migration/

(refugee? or immigrant?).ab,kw,ti.

or/4-9 [race, minorities and ethnicity]

health care policy/ or "attitude to health"/ or patient participation/ or symbolism/

((self or social or cultural or donor?) adj2 identity).ab,kw,ti

(behavio?r or motivation or psychol* or psychiatr* or attitude? or altruism or volunteer* or representation? or barrier? or facilitator? or antecedent? or belief? or value? or norm? or intention* or self efficacy or willing* or unwilling* or taboo? or cultural divers* or cultural characteristic? or superstition? or policy or policies or distance or deterrent? or environmental factor? or location or literacy or (ear* adj3 (experience or history)) or demographic or family or knowledge or education or motivation or demotivation or socio* or accessib* or inaccessib* or trust* or distrust* or mistrust* or relig* or faith or believe? or stigma or discriminat* or aware* or anxiety or fear or recruitment or retention or external factor* or determinant? or myth? or folklore or symbolism).ab,kw,ti

((perspective? or perception?) adj5 (patient? or donor? or donation or respondent? or participant? or client?)).ab,kw,ti

(increas* adj3 (donor? or donation)).ab,kw,ti

or/11-15 [factors]

3 and 10 and 16

(animal/ or animal experiment/ or animal model/ or nonhuman/ or rat/ or mouse/ or (rat or rats or mouse or mice).ti.) not human/

17 not 18

=================

**20151019 PsycINFO**

tissue donation/

(blood adj2 don*).ab,id,ti.

or/1-2 [blood]

exp ethnic group/ or exp ancestors/ or minority health/ or minority group/ or "racial and ethnic differences"/ or diversity/ or exp "racial and ethnic groups"/ or ethnic identity/ or cultural sensitivity/ or ethnic values/

(race or racial or ethnic* or ethno* or transients or minorities or minority group? or cultural sensitivity).ab,id,ti.

((origin? or communit* or descent or descendant or heritage) adj5 (subsaharan or "sub saharan" or african or south american or carribean or non european)).ab,id,ti.

(non white? or nonwhite? or nonwestern or non western or nonenglish or non english or blacks or afro or afroamerican*).ab,id,ti.

exp migrant/ or exp human migration/

(refugee? or immigrant?).ab,id,ti.

or/4-9 [race, minorities and ethnicity]

exp personality traits/ or exp religious beliefs/ or false beliefs/ or health care policy/ or symbolism/ or health attitudes/ or health education/ or health knowledge/ or client participation/ or health behavior/ or knowledge level/

(recruitment or retention or external factor* or determinant? or myth? or folklore or symbolism or attitude? or altruism or volunteer* or representation? or barrier? or facilitator? or antecedent? or belief? or value? or norm? or intention* or self efficacy or willing* or unwilling* or taboo? or cultural divers* or cultural characteristic? or superstition? or policy or policies or distance or deterrent? or environmental factor? or location or literacy or (ear* adj3 (experience or history)) or demographic or family or knowledge or education or motivation or demotivation or socio* or accessib* or inaccessib* or trust* or distrust* or mistrust* or relig* or faith or believe? or stigma or discriminat* or aware* or anxiety or fear).ab,id,ti

exp client attitudes/ or exp self concept/

((perspective? or perception?) adj5 (patient? or donor? or donation or respondent? or participant? or client?)).ab,id,ti

((self or social or cultural or donor?) adj2 identity).ab,id,ti

(increas* adj3 (donor? or donation)).ab,id,ti

(2920 or 2930 or 3000 or 3020 or 3040).cc

or/11-17 [factors]

3 and 10 and 18

limit 19 to human

===============

**20151019 BIOSIS**

(blood adj2 don*).ab,kw,ti.

(race or racial or ethnic* or ethno* or transients or minorities or minority group? or cultural sensitivity).ab,mi,ti.

((origin? or communit* or descent or descendant or heritage) adj5 (african or south american or middle eastern or carribean or subsaharan or "sub saharan")).ab,mi,ti.

(non white? or nonwhite? or nonwestern or non western or nonenglish or non english or blacks or afro or afroamerican* or non european).ab,mi,ti.

(refugee? or immigrant?).ab,mi,ti.

or/2-5 [race, minorities and ethnicity]

((self or social or cultural or donor?) adj2 identity).ab,mi,ti

(behavio?r or motivation or psychol* or psychiatr* or attitude? or altruism or volunteer* or representation? or barrier? or facilitator? or antecedent? or belief? or value? or norm? or intention* or self efficacy or willing* or unwilling* or taboo? or cultural divers* or cultural characteristic? or superstition? or policy or policies or distance or deterrent? or environmental factor? or anxiety or fear or accessib* or inaccessib* or trust* or distrust* or mistrust* or relig* or faith or believe? or stigma or discriminat* or aware* or location or literacy or (ear* adj3 (experience or history)) or demographic or family or knowledge or education or motivation or demotivation or socio* or recruitment or retention or external factor* or determinant? or myth? or folklore or symbolism).ab,mi,ti

((perspective? or perception?) adj5 (patient? or donor? or donation or respondent? or participant? or client?)).ab,mi,ti

(increas* adj3 (donor? or donation)).ab,mi,ti

or/7-10 [factors]

1 and 6 and 11

Appendix B

*Quality criteria and full assessment of quantitative studies*

**Did the study address a clearly focused issue? (Singh, 2013)**

**Specific objectives described? (Von Elm, Altman *et al.*, 2007)**

Population (Singh, 2013)

Factors

Outcome

**Was the study design appropriate to address the aims of the research? (Law, Stewart *et al.*, 1998; Kmet, Lee *et al.*, 2004)**

**Was the cohort recruited in an acceptable way? (Singh, 2013)**

Was the cohort representative of a defined population? (Singh, 2013)

Was the setting and location well described? (Von Elm, Altman *et al.*, 2007)

Periods of recruitment? (Von Elm, Altman *et al.*, 2007)

Are the eligibility criteria described? (Von Elm, Altman *et al.*, 2007)

**Were variables accurately measured? (Singh, 2013)**

Was the outcome (blood donation; behaviour or intention) well defined? (Singh, 2013)

Were factors (barriers and facilitators) to blood donation well defined? (Singh, 2013)

Were the outcome measurements validated? (Singh, 2013)

Were the factors measured with validated measurements? (Singh, 2013)

**Analysis described/justified and appropriate? (Kmet, Lee *et al.*, 2004; Singh, 2013)**

Were all statistical methods described? (Von Elm, Altman *et al.*, 2007)

Did they used the correct statistical method to answer the research question?

Were relevant confounders taken into account for blood donation (age, sex, education level, race, donor status)? (Singh, 2013)

Sample size considered? (Law, Stewart *et al.*, 1998; Von Elm, Altman *et al.*, 2007)

**Results reported in sufficient detail and reliable? (Singh, 2013)**

Response rate and characteristics of the study population described?

What is the confidence interval, if given? (Law, Stewart *et al.*, 1998)

Are the statistical analyses and results reported correctly and completely?

Were the results correctly interpreted?

**Is impact of the research discussed?**

Were the research questions/hypotheses answered?

Are implications for practice and further research described? (Law, Stewart *et al.*, 1998; Singh, 2013)

Comparison with other studies?

Conclusions supported by the results? (Kmet, Lee *et al.*, 2004)

Are limitations described? (Law, Stewart *et al.*, 1998; Von Elm, Altman *et al.*, 2007)

Appendix C
*Quality criteria and full assessment of qualitative studies*

**Was there a clear statement of the aims of the study? (Singh, 2013)**

Was the goal of the study described? (Singh, 2013)

Was the relevance of the study described? (Offringa, Assendelft *et al.*, 2003; Singh, 2013)

**Was the research design appropriate to address the aims of the study? (Offringa, Assendelft *et al.*, 2003)**

Is discussed why this design method is chosen? (Singh, 2013)

Did the researchers seek to interpret or illuminate the actions and/or subjective experiences of the participants (rather qualitative than quantitative)? (Singh, 2013)

**Was the research connected to a theoretical framework and/or wider body of knowledge? (Kmet, Lee *et al.*, 2004)**

**Was the recruitment- of information assemblage strategy described and justified? (Offringa, Assendelft *et al.*, 2003; Kmet, Lee *et al.*, 2004)**

Inclusion/exclusion criteria described (of persons/information sources)?

Is explained why these persons/information sources are the most appropriate for this study? (Singh, 2013)

Is the setting of data collection described? (Tong, Sainsbury *et al.*, 2007)

Has the researchers critically examined their own role, potential bias and/or influence in this process? (Tong, Sainsbury *et al.*, 2007; Singh, 2013)

Have ethical issues been taken into consideration? (Mills, Jadad *et al.*, 2005; Singh, 2013)

**Was the data collection methods clearly described and systematic? (Kmet, Lee *et al.*, 2004)**

Is it clear how data were collected? (Singh, 2013)

Were data transcribed verbatism (e.g. were audiotapes, videotapes, or field notes used)? (Offringa, Assendelft *et al.*, 2003; Mills, Jadad *et al.*, 2005)

If applicable, were (interview) questions predefined? (Mills, Jadad *et al.*, 2005)

Are verification procedures done (e.g. peer reviews, member checks)? (Kmet, Lee *et al.*, 2004; Mills, Jadad *et al.*, 2005)

**Was there a clear statement of findings? (Singh, 2013)**

Is there an in-depth description of the analysis process? (Offringa, Assendelft *et al.*, 2003; Singh, 2013)

Do sequences/quotations from the original data support the results? (Offringa, Assendelft *et al.*, 2003; Mills, Jadad *et al.*, 2005; Tong, Sainsbury *et al.*, 2007)

Were research themes clearly presented in the findings? (Kmet, Lee *et al.*, 2004)

Was evidence both for and against the researcher's argument discussed? (Offringa, Assendelft *et al.*, 2003; Singh, 2013)

**How valuable is the research? (Singh, 2013)**

Are conclusions supported by the results? (Kmet, Lee *et al.*, 2004; Singh, 2013)

Are limitations described?

Are new areas for further research identified and discussed? (Singh, 2013)

**Appendix references**

Kmet, L.M., R.C. Lee, et al. (2004). Standard quality assessment criteria for evaluating primary research papers from a variety of fields., 1–20. Alberta Heritage Foundation for Medical Research (AHFMR), (HTA Initiative #13).

Law, M., D. Stewart, et al. (1998). Critical review form–quantitative studies. *McMaster University: Occupational Therapy Evidence-Based Practice Research Group*.

Mills, E., A.R. Jadad, et al. (2005). Systematic review of qualitative studies exploring parental beliefs and attitudes toward childhood vaccination identifies common barriers to vaccination. *Journal of clinical epidemiology* **58**, 1081-1088.

Offringa, M., W.J.J. Assendelft, et al. (2003). *Inleiding in evidence-based medicine. Klinisch handelen gebaseerd op bewijsmateriaal*, Houten.

Singh, J. (2013). Critical Appraisal Skills Programme. *Journal of Pharmacology and pharmacotherapeutics* **4**, 76.

Tong, A., P. Sainsbury, et al. (2007). Consolidated criteria for reporting qualitative research (COREQ): a 32-item checklist for interviews and focus groups. *International Journal for Quality in Health Care* **19**, 349-357.

Von Elm, E., D.G. Altman, et al. (2007). The Strengthening the Reporting of Observational Studies in Epidemiology (STROBE) statement: guidelines for reporting observational studies. *Preventive medicine* **45**, 247-251.
